# The soluble guanylate cyclase activator BAY 58-2667 protects against morbidity and mortality in endotoxic shock by recoupling organ systems

**DOI:** 10.1186/2050-6511-14-S1-P75

**Published:** 2013-08-29

**Authors:** Benjamin Vandendriessche, Elke Rogge, Vera Goossens, Peter Vandenabeele, Johannes-Peter Stasch, Peter Brouckaert, Anje Cauwels

**Affiliations:** 1 Department for Molecular Biomedical Research, VIB, Ghent, Belgium; 2 Department of Biomedical Molecular Biology, Ghent University, Ghent, Belgium; 3 Institute of Cardiovascular Research, Bayer HealthCare, Wuppertal, Germany

## Background

Sepsis and septic shock are associated with high mortality rates and the majority of sepsis patients die due to complications of multiple organ failure (MOF). The cyclic GMP (cGMP) producing enzyme soluble guanylate cyclase (sGC) is crucially involved in the regulation of (micro)vascular homeostasis, cardiac function and, consequently, organ function. However, it can become inactivated when exposed to reactive oxygen species (ROS). The resulting heme-free sGC can be reactivated by the heme- and nitric oxide (NO)-independent sGC activator BAY 58-2667 (Cinaciguat).

## Results

We report that late (+8h) post-treatment with BAY 58-2667 in a mouse model can protect against lethal endotoxic shock. Protection was associated with reduced hypothermia, circulating IL-6 levels, cardiomyocyte apoptosis, and mortality. In contrast to the beneficial effect of BAY 58-2667, the sGC stimulator BAY 41-2272 and the phosphodiesterase 5 inhibitor Sildenafil did not have any beneficial effect on survival, emphasizing the importance of the selectivity of BAY 58-2667 for diseased vessels and tissues. Hemodynamic parameters (blood pressure and heart rate) were decreased, and linear and nonlinear indices of blood pressure variability, reflective for (un)coupling of the communication between the autonomic nervous system and the heart, were improved after late protective treatment with BAY 58-2667.

## Conclusion

In conclusion, our results demonstrate the pivotal role of the NO/sGC axis in endotoxic shock. Stabilization of sGC function with BAY 58-2667 can prevent mortality when given in the correct treatment window, which probably depends on the dynamics of the heme-free sGC pool, in turn influenced by oxidative stress. We speculate that, considering the central role of sGC signaling in many pathways required for maintenance of (micro)circulatory homeostasis, BAY 58-2667 supports organ function by recoupling inter-organ communication pathways.

